# Pickering Emulsions Stabilized by Hybrid TiO_2_-pNIPAm Composites for the Photocatalytic Degradation of 4-Propylbenzoic Acid

**DOI:** 10.1021/acsomega.4c07847

**Published:** 2025-01-07

**Authors:** Zygimantas Gricius, Gisle Øye

**Affiliations:** Ugelstad Laboratory, Department of Chemical Engineering, Norwegian University of Science and Technology (NTNU), 7491 Trondheim, Norway

## Abstract

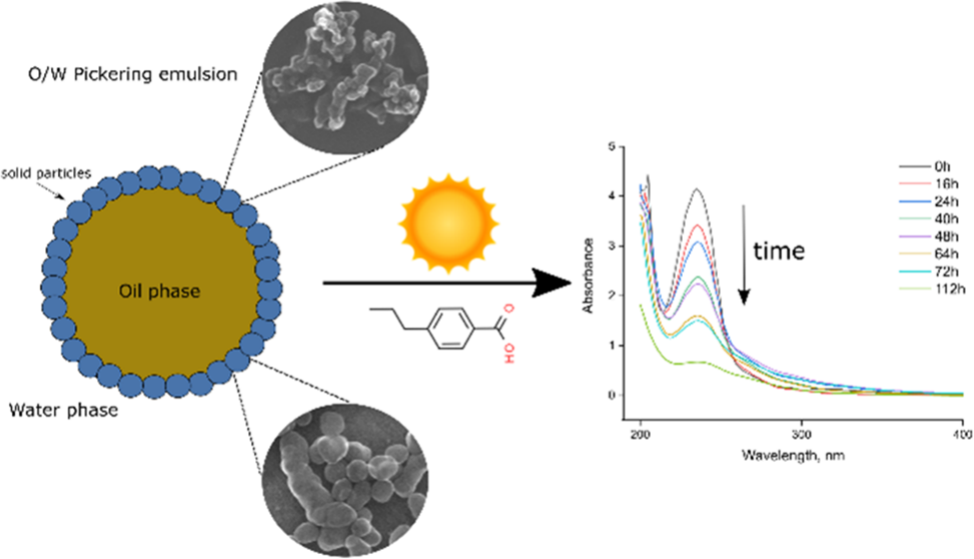

Pickering emulsions (PEs) have demonstrated significant potential in various fields, including catalysis, biomedical applications, and food science, with notable advancements in wastewater treatment through photocatalysis. This study explores the development and application of TiO_2_-poly(*N*-isopropylacrylamide) (pNIPAm) composite gels as a novel framework for photocatalytic wastewater remediation. The research focuses on overcoming challenges associated with conventional nanoparticle-based photocatalytic systems, such as agglomeration and inefficient recovery of particles. By integrating TiO_2_ nanoparticles into pNIPAm gels, we aimed to achieve high emulsion stability and photocatalytic efficiency while suppressing the effects of pNIPAm’s volume phase transition temperature (VPTT) to facilitate effective emulsion recovery. The study involves the synthesis of TiO_2_-pNIPAm composites with varying monomer-to-particle ratios, characterizing their VPTT behavior, morphology, and thermal stability. These composites were then evaluated for their emulsification properties, phase transition behavior, and photocatalytic activity in degrading 4-propylbenzoic acid, a model pollutant. The results highlight the effectiveness of the TiO_2_-pNIPAm Pickering emulsions in wastewater treatment, offering improved stability and reusability compared to traditional dispersion-based systems. This work provides new insights into the design of composite materials for enhanced photocatalytic applications and demonstrates the potential of Pickering emulsions in sustainable environmental remediation.

## 1 Introduction

Pickering emulsions (PEs) have been studied within a broad range of applications, including catalysis,^1^ biomedical applications^2^ and food science.^3^ They have also proven to be a versatile framework employed in crafting functional materials applicable for diverse wastewater treatment methods.^4^ This potential has been showcased using PEs in photocatalysis, adsorption, membrane filtration, and chemical disinfection, all of which contribute to effective pollutant degradation. Among the tested approaches, Pickering emulsion-based photocatalysis has emerged as a technology with multiple case studies showing high photodegradation efficiency and easy recovery of emulsions.^5,6^ Moreover, their distinctive characteristics, including a large surface area, stability, ease of production, and adjustability, render them highly appealing for industrial applications. In typical dispersion-based photocatalytic systems, nanoparticles are often prone to agglomeration resulting in an uneven particle distribution and limited surface area for the photocatalysis.^6^ Moreover, traditional methods of collecting the photocatalysts, such as centrifugation or filtration, can be energy and cost intensive. Although not directly preventing particle aggregation at the droplet surface, Pickering emulsions provide improved stability toward coalescence and phase separation. This is particularly useful in commercial uses, where the recovery and reuse of catalysts are essential for economic and environmental reasons. As a result, integration of the favorable characteristics of nanoparticle dispersions with the advantages of Pickering emulsions — such as providing large active catalyst areas and the potential for catalyst recovery through emulsion collection and reuse — puts particle-stabilized emulsions into perspective as promising candidates for the photocatalytic remediation of wastewater streams.^1,7^

Until now, most studies have relied on improving the photocatalytic efficiency of PEs by varying the particle size, shape, and composition of nanoparticles to maximize light absorption and catalytic activity. However, hydrophilic nanoparticles typically require wetting agents to facilitate adsorption at the oil–water interface.^8,9^ This is often achieved using surfactants, which can degrade over time, thereby compromising the long-term usability of such systems.^10,11^ As an alternative, high adsorption was shown to be achieved by tuning the wettability of nanoparticles through the creation of polymer-inorganic oxide composites.^12,13^ However, several studies have reported high stability of pNIPAm-stabilized emulsions owing to the hydrogel’s intermediate wetting properties with an improved monolayer homogeneity at lower particle sizes making it an interesting Pickering emulsifier to explore in conjunction with photocatalytic nanoparticles.^14−16^ Nanogels (NG) based on poly(*N*-isopropylacrylamide) (pNIPAm), characterized by their ability to undergo a reversible volume phase transition in response to changes in temperature, provide a unique and dynamic interface for interaction with inorganic oxides.^17−19^ This responsiveness, driven by the volume phase transition temperature (VPTT) behavior of pNIPAm, enables precise control over the composite’s structure, properties, and functionality in response to external stimuli. However, for photocatalytic Pickering emulsions, the VPTT behavior limits emulsion recovery, as changes in the particle size result in droplet coalescence and phase separation. Therefore, the goal here was to suppress the VPTT changes of pNIPAM gels while retaining its wetting properties, which would give rise to stable emulsions even under irradiation. This would allow efficient emulsion recovery, providing a clear advantage over the dispersion-based photocatalytic systems.

Among the wide range of photocatalysts, titania was chosen in the study owing to its stability, low cost, and nontoxic nature.^20^ However, previous studies indicate that stabilizing Pickering emulsions using only titania nanoparticles is challenging.^21−23^ While there have been multiple instances of grafting pNIPAm to titania, this has generally been done by incorporating carboxylic acid or sulfonate - based comonomers, which facilitate the grafting of inorganic oxide surfaces to the polymer matrix.^17,24−26^ However, there have not been reports using such composites in Pickering emulsions. Additionally, production of Pickering emulsifiers necessitates deviations from the conventional synthetic route, as incorporating a charged comonomer in the polymerization mixture would jeopardize the resulting composite’s role as a Pickering emulsifier owing to disruptions of the hydrophilic/hydrophobic balance.^27,28^ The free radical polymerization of pNIPAm gels is known to be influenced by many factors, including reactant concentrations, surfactant concentration, initiator amount, and reaction temperature, but little is known about the effects coming from incorporation of solid-oxide nanoparticles, like titania.^17,29,^ Therefore, the impact of nanoparticle loading to wettability and the VPTT values needs to be addressed when designing composite systems for photocatalytic applications.

In this work, we aimed to produce and characterize model Pickering emulsion-based photocatalytic degradation systems by employing TiO_2_-pNIPAm composite gels with the gel part used as a carrier for the immobilized titania nanoparticles onto the oil–water interface. The produced composites were characterized in terms of their VPTT behavior, morphology and thermal decomposition. A section on the synthesis was included to analyze the influence of dispersed particles on the composite particle size and zeta potential. Different variations of the composites were produced by tuning the mass ratio of NIPAm to TiO_2_, with a focus on the emulsification and phase transition behavior. These properties were predicted to be decisive for the photocatalytic activity and composite recovery purposes. The stability of the particles as Pickering emulsifiers was assessed by producing Pickering emulsions and subjecting them to model wastewater solutions for extended periods of irradiation by a solar simulator. The potential of the produced emulsions as photocatalytic wastewater remediation systems was evaluated on the basis of the photocatalytic degradation of a model pollutant, 4-propylbenzoic acid, which is a naphthenic acid (NA) commonly found in produced water and known to produce adverse effects on aquatic ecosystems.^31^ Finally, reusability of the produced emulsions was assessed to showcase the potential of photocatalytic Pickering emulsions against dispersion-based solutions in wastewater treatment.

## 2 Experimental Section

### 2.1 Materials

Titanium(IV) oxide (TiO_2_, ≥99.5% trace metals basis, 21 nm primary particle size), *N*-isopropylacrylamide (NIPAm, 97%), *N*–*N*′-methylene-bis(acrylamide) (BIS, 99%), sodium dodecyl sulfate (SDS, 99%), potassium persulfate (KPS, 99%), trisodium citrate dihydrate (analytical grade), silicone oil (5 cSt), 4-propylbenzoic acid (4-pb) and dialysis tubing (cellulose, MWCO 14,000, 99.99% retention) were purchased from Sigma–Aldrich (Schnelldorf, Germany) and used without further purification or modification. Water used in the experiments was purified using a Simplicity Millipore (Darmstadt, Germany) water purification system. The recorded resistivity of water for all the procedures was 18.2 MΩ·cm at 25 °C.

### 2.2 Stabilization of TiO_2_ Nanoparticles

Pristine TiO_2_ nanoparticles came in a flocculated state and exhibited poor dispersion stability in the monomer solution, therefore additional dispersion and stabilization steps were required prior to using them in the synthesis of pNIPAm/TiO_2_ composites. First, TiO_2_ powder was dispersed in 1 mM trisodium citrate solution resulting in a titania concentration of 4 mg/mL. Then, an ultrasonic homogenizer (Fisherbrand Model 505, 2 min, 2s pulse mode, 40% amplitude) was used to produce a stable dispersion of clustered titania around 200 nm in diameter.

### 2.3 Synthesis of pNIPAm/TiO_2_ Composites

**Figure 1:**
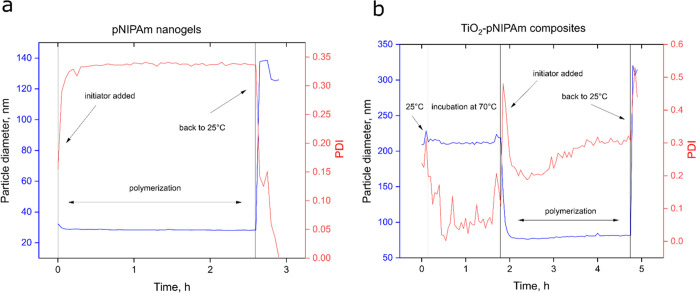
Real-time particle size (blue) and polydispersity (red) monitored during the polymerization reaction for selected formulations: (a) bare gel (NG), (b) 0.1Ti-100.

**Scheme 1:**
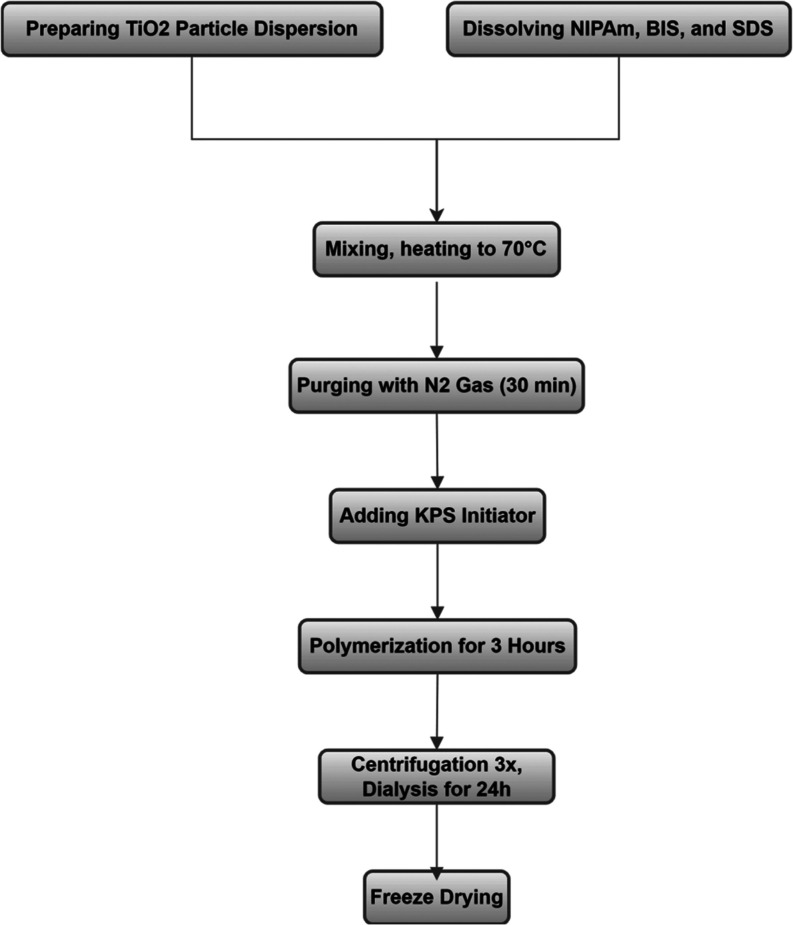
Flow Chart for the Synthesis of TiO2-pNIPAm Composites

The pNIPAm/TiO_2_ composites were synthesized using in situ precipitation polymerization with titania nanoparticles dispersed in the vials before the start of polymerization. NIPAm and BIS were used as the monomer and cross-linker, respectively; SDS served as the surfactant, and the reaction was initiated by introducing KPS. In a typical reaction, a fixed amount of titania from the previously stabilized dispersion was added to a 250 mL round-bottom flask where it was diluted with SDS to obtain the required concentration (Table 1). The naming convention XTi-Y states the starting concentration of titania (X) with the corresponding mass ratio of NIPAm to TiO_2_ (Y). The standard sample volume was 80 mL, although scaled down variants were produced as well. For example, during the real-time synthesis monitoring (Figure 1), the synthesis was performed directly in a closed cuvette by scaling down the precursor amounts to 3 mL total volume. The final concentration of SDS was adjusted to 4 mM. Then, both NIPAm and BIS were added, and the mixture was heated to 70 °C under stirring. Next, the solution was subject to vigorous flushing with N_2_ gas for 30 min. After 30 min, KPS was added to the mixture and the flushing was continued for another 15 min to remove the air introduced during the addition of KPS. Finally, the reaction continued for 3h at 70 °C under stirring. All the synthesized NGs were dialyzed thrice within 24 h to remove any unreacted monomers and residual reactants. For the composite characterization beyond the polymerization analysis, the samples were additionally washed several times with MQ water after centrifugation at 15,000 rpm for 1h in order to remove the free gels, the resulting samples were redispersed in MQ water. The purified composites were freeze-dried (VirTis BenchTop Pro with Omnitronics, SP Scientific, Stone Ridge, NY) to remove the solvent and stored at room temperature for further analysis and the photocatalytic degradation studies. The hybrid gel preparation workflow is additionally shown in Scheme 1.

**Table 1:** pNIPAm-TiO_2_ Compositions by Varying the Starting TiO_2_ Concentration and the Mass Ratio of NIPAm to TiO_2_

**formulation**	**c(TiO**_**2**_**)**,mg/mL	**c(NIPAm)**,mg/mL	**mass ratio(NIPAm to TiO_2_)**	**weight ratio(BIS to monomer)**	**c(KPS)**, mg/mL
NG	0	10	0	0.05	0.625
0.5Ti-0.2	0.5	0.1	0.2	0.05	0.625
0.5Ti-0.5	0.5	0.25	0.5	0.05	0.625
0.5Ti-5	0.5	2.5	5	0.05	0.625
0.5Ti-10	0.5	5	10	0.05	0.625
0.5Ti-20	0.5	10	20	0.05	0.625
0.1Ti-25	0.1	2.5	25	0.05	0.625
0.1Ti-50	0.1	5	50	0.05	0.625
0.1Ti-100	0.1	10	100	0.05	0.625

### 2.4 Composite Characterization

The freeze-dried samples were characterized in Fourier transform infrared (FT-IR) spectroscopy analysis using a Bruker Tensor 27 FT-IR spectrophotometer equipped with a single reflection Attenuated Total Reflection (ATR) cell containing a diamond crystal (MKII Golden Gate, Specac). For the catalyst morphology examination, a Hitachi SU9000 Scanning Transmission Electron Microscope (STEM) was employed. Both secondary electron (SE) and dark-field transmission (DF-TEM) images were captured at an acceleration voltage of 30 kV and a current of 15 μA. To prepare the samples for ST(E)M, a diluted suspension of the catalyst was drop-cast onto a TEM grid (Formvar/Carbon 300 Mesh, Cu), which was then sputter coated (Cressington 208 HR B) with Pt/Pd (5 nm thickness, 80/20) in order to improve the sample conductivity. The particle sizes and zeta potentials of the particles were determined using a Malvern Zetasizer Nano-ZS, with a 2 min equilibration period for the samples before the measurements were initiated. The droplet size distribution of the produced emulsions was characterized by using both optical microscopy (Nikon Eclipse ME600) and a laser diffraction particle sizer – Mastersizer 3000 (Malvern). For each condition, three sample runs were captured and the average distribution was calculated. For the droplet size distribution measurements, the samples had to be diluted multiple times to attain the necessary obscuration level for emulsions (10–20%).

### 2.5 Volumetric Phase Transition Temperature (VPTT) and Volumetric Collapse (VC) Calculation

VPTT of the composites was calculated by plotting the size evolution of the particles over a temperature range from 25 to 46 °C.^32^ The sizes of these composites were measured between 25 and 46 °C with a varying interval from 1 to 3 °C. A well-known phase transition, due to particle collapse, was recorded with increasing temperature as indicated by sigmoidal particle size evolution curves.

In order to describe the phase behavior, the change in the particle size of the composites was normalized according to eq 1:
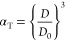
1where, *D* is the hydrodynamic diameter at a given temperature and *D*_0_ is the diameter at 25 °C. Then, a sigmoidal 5 parameter fitting (eq 2) was performed by using SigmaPlot version 14.0. The obtained fitting parameters were used to calculate the VPTT by equating the areas under the heating curves as described by Bandyopadhyay et al.^32^
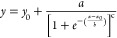
2Similarly, the volumetric collapse of the composites was assessed by taking the hydrodynamic diameter values at the initial (25 °C) and final (46 °C) temperatures according to eq 3.
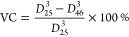
3

### 2.6 Emulsion Preparation

To make Pickering emulsions with the produced composites as stabilizers, Ika Werke Ultra Turrax T25 homogenizer with a S25 N-8 G dispersing tool was used. Typically, 10% w/w silicone oil in water emulsions were produced by weighing 18 g of the water phase (dispersed particle composites) and 2 g of the oil. Silicone oil was selected as an inert oil phase, which could not further be oxidized at the oil–water interface during photocatalysis. The mixture was then homogenized for 2 min at 20,000 rpm. The resulting emulsions were found to be oil-in-water by performing a drop test–a drop of emulsion homogeneously distributed upon dilution in an aqueous phase. For the partitioning experiments, the prepared emulsions were aged for 48 h. Then, the upper oil and emulsion layers were carefully separated from the water phase. One mL of each sample was retrieved from the water phase, diluted 10 times and analyzed by UV–vis. The resulting particle concentrations were determined by interpolating the absorbance values from the calibration curves, which were previously made from the equivalent dispersions.

### 2.7 Photocatalytic Efficiency Testing

The photocatalytic efficiency of the produced composites was evaluated by using a solar simulator (SCIENCETECH SF300-A) equipped with a 300 W xenon arc lamp and AM1.5G filter. The spectral irradiance of the solar simulator is shown in Figure S1. Additionally, a band gap analysis of bare titania nanoparticle dispersions was conducted by extracting Tauc plots from the UV–vis absorption data (Figure S2). This analysis aimed to identify the modes of electronic transitions and illustrate the overlap of TiO_2_ NP absorbance spectra with the intensity of irradiated light from the solar simulator. According to the figures, both direct and indirect band gap energies were determined to be 3.46 eV (358 nm) for direct and 2.55 eV (488 nm) for indirect transitions. The reported value for bulk TiO_2_ varies from 3.0 to 3.2 eV depending on the crystalline phase; however, increase in energy is expected for nanoparticles due to the electron confinement effect.^33,34^ As a result, the photodegradation is expected to be driven by the indirect band gap transitions since only the indirect region overlaps with the spectrum of the solar simulator.

In a typical experiment, 20 g of 0.05 mg/g (by TiO_2_) TiO_2_–NG emulsion was produced by mixing a 0.1 mg/g prior made emulsion with a 4-pb solution in 25 mM borax (pH 9) buffer. The standard 4-pb concentration in a test vial was 3 mM. The amount of titania was kept constant for the different gel formulations. The diluted emulsion with 4-pb is then placed in front of the aperture of the solar simulator at a distance, where the precalibrated light intensity equals 1 sun (100 mW/cm^2^). Then, 1 mL aliquots were taken at different timestamps at a frequency of two samples per day (9 am and 4 pm) for 112 h. The aliquots were then centrifuged at 14,500 rpm for 10 min using an Eppendorf MiniSpin Plus centrifuge followed by supernatant collection with a 1 mL syringe via 0.22 μm cellulose acetate filter. In a separate experiment, the adsorption of 4-pb by the syringe filter was found to be negligible. Then, 300 μL of the supernatant was taken to be diluted up to 3 mL in a quartz cuvette. The absorbance of the supernatant was acquired using a Cary 3500 Multicell UV–vis (Agilent) spectrophotometer. The spectra were collected over a range from 200–800 nm and the absorbance at 235 nm was used to estimate the degradation of 4-pb based on the previously acquired calibration curve (Figure S3). The efficacy of the test particles was compared with respect to both pure titania-stabilized emulsions and 4-pb without nanoparticles acting as the control sample.

## 3 Results and Discussion

### 3.1 TiO_2_/pNIPAm Synthesis

In order to analyze the particle growth mechanism in the presence of titania nanoparticles, the polymerization reaction was monitored by following the real-time changes of the particle diameter and the corresponding polydispersity (PDI) values. The evolution of hydrodynamic diameters depicted in Figure 1a,b illustrates the growth of the pNIPAm gels with and without titania dispersed in the water phase. To gain insight into the growing mechanism, the composite formulation with the lowest amount of titania was studied. As seen from Figure 1a, there was a rapid increase in the PDI within the first 3 min after adding the initiator up until it reached a plateau value of 0.34 ± 0.00. The particle size during the polymerization step did not change significantly and revolved around 28 nm. During the polymerization, the growing chains were above their VPTT temperature, therefore the collapse on the primary seed particles occurred immediately. The particle size control was ensured by SDS adsorbed on the gels forming a stabilizing layer which prevented the aggregation or coalescence of the gel particles.^17^ After the mixture was cooled down to 25 °C, there was a substantial increase in particle size up to 126 ± 1 nm, which is expected for pNIPAm hydrogels below their VPTT temperature. Overall, the produced gels were found to be highly monodisperse.

Similar behavior was seen for the composite (Figure 1b), where the surface for nucleation was provided before the start of polymerization. The measurement started at 25 °C with citrate-stabilized titania having a particle size of 212 ± 3 nm, which were dispersed in a mixture containing both the monomers and SDS. Then, the temperature was increased to 70 °C with no significant changes in particle size. The mixture was kept for 1.5 h at 70 °C to observe any changes associated with the primary titania particles. Finally, the initiator was added to the mixture resulting in a sharp increase in polydispersity and decrease in the particle diameter. This effect was associated with the creation of contracted gel particles driving the average particle diameter to lower values. During the course of polymerization there was a gradual increase in polydispersity and particle diameter, which was expected for pure titania being incorporated into the gel composites. Finally, as the mixture was cooled down, the particle size increased resembling the previously observed pattern in the bare nanogel synthesis. The final particle size resembled a sum of clustered titania and the size of the bare nanogels.

**Figure 2:**
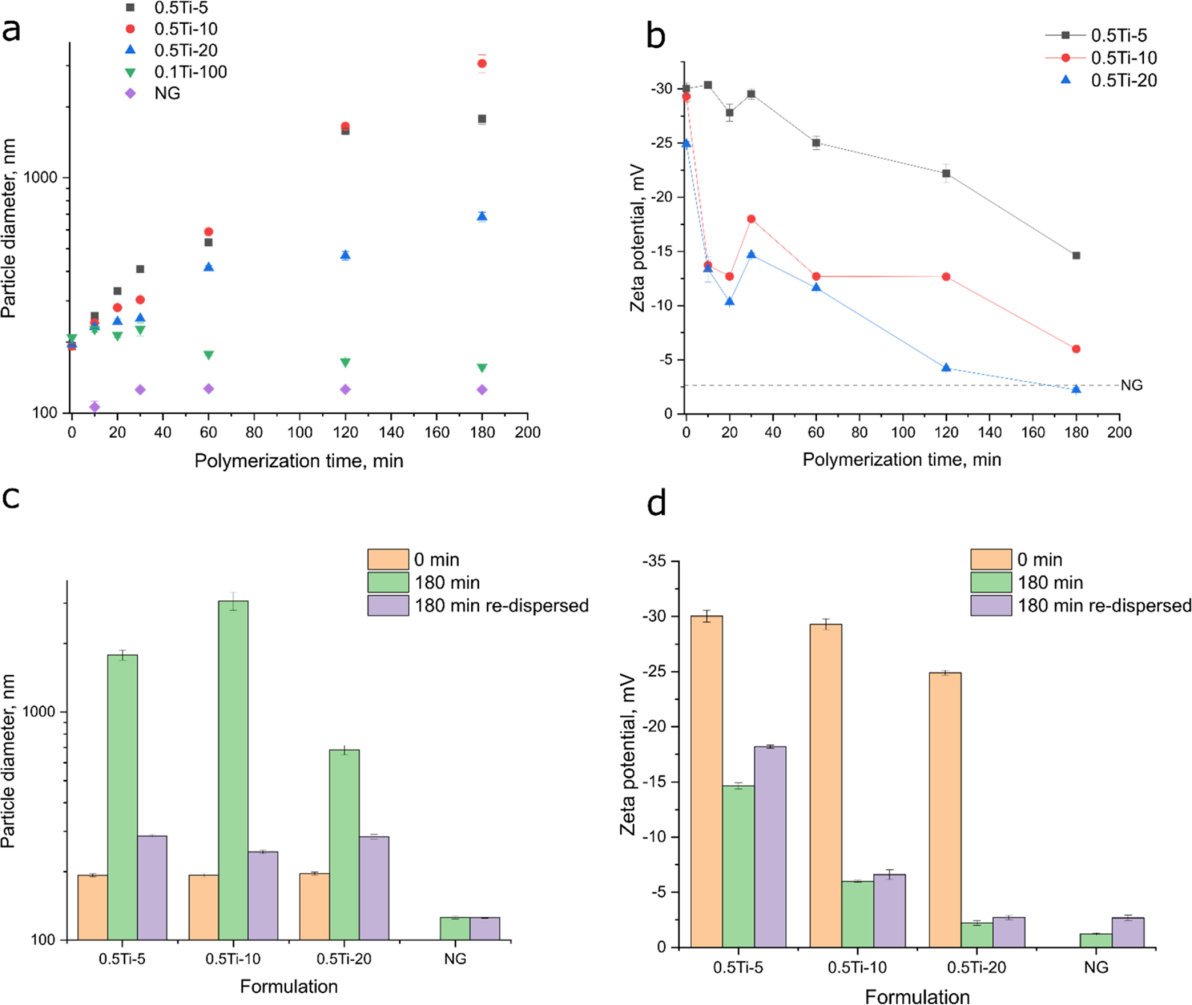
Variation of particle diameter (a) and zeta potential (b) at different polymerization times from the aliquots at room temperature; the corresponding values for the samples including the values after redispersion by ultrasonication (c and d).

Since the polymerization took place at temperatures above the VPTT of pNIPAm, it was not possible to estimate equilibrium particle size and zeta potential at room temperature as a function of polymerization time. To address this issue, fractions of polymerization mixture were taken at different timestamps in order to observe the evolution of these parameters below the VPTT (Figure 2a,b). As indicated by Figure 2a, there was a gradual increase in the particle size during the polymerization for the composite particles resulting in the final composite particles in the range of a few micrometers, whereas the free nanogels reported a stable size of around 120 nm already after 30 min in the process. Given that both free titania and nanogels did not change their dimensions after 30 min, the increase in composite size suggests an interaction between TiO_2_ and pNIPAm, which leads to aggregated composites. Interestingly, the NIPAm-rich composite (0.1Ti-100) showed a dual behavior in particle size with an initial rise within the first 30 min followed by a gradual decrease in size gradually converging to the value of the free nanogel solution. This can be viewed as a model system showing composite formation initially (0–30 min) followed by the generation of free nanogels due to NIPAm being in large excess. In this case, the proposed stacking of hybrid nanogels was not observed, which may indicate changes in morphology between the TiO_2_-rich and NIPAm-rich samples. The presence of free nanogels in 0.1Ti-100 was additionally confirmed by measuring the particle size of the supernatant after centrifugation, which matched the value of the pure nanogel. To supplement the findings from the particle size data, evolution of the ζ potential with respect to polymerization time was monitored. In the 0.1Ti-100 case, a sharp decrease in the zeta potential was noted after 10 min into polymerization, which then stabilized at the value of the free nanogel at 180 min matching the observed trends in the particle size data (Figure S4). In comparison, a much smaller drop in the zeta potential was found for the TiO_2_-rich formulations (Figure 2b) with values at 180 min for both 0.5Ti-5 and 0.5Ti-10 remaining above the free nanogels’ reference line. These formulations were considered to have far fewer free nanogels with the titania particles decorating the surface of the gels. After carrying out centrifugation, the supernatant was found to be completely transparent compared to the free nanogel sample indicating a successful binding between pNIPAm and titania. In contrast, similar analysis of 0.5Ti-20 revealed that the sample contains substantially more free nanogels, which was also supported by lower zeta potential and particle size values at 180 min. The revealed pattern gave insight into the composite growth mechanism and suggested an optimization route by increasing the relative amount of TiO_2_ with respect to the monomer, which would likely result in a higher loading of titania with less free nanogels. A more detailed representation of the composite structures is shown in the later sections.

With the particle size reported in the range of a few micrometers, the TiO_2_-rich samples were further analyzed to gain insight into the growth mechanism. The samples were cooled down to room temperature and redispersed by ultrasonication, which then led to a drastic reduction in the particle size (Figure 2c) for all but the free nanogel samples. This observation supported the theory of composite stacking, which occurs when the titania particles are exposed on the surface of the hydrogels effectively acting as a weak cross-linking agent. In the absence of comonomers, the interaction between titania and pNIPAm is expected to be governed by hydrogen bonding and van der Waals forces closely resembling the interactions with water molecules via C=O and N–H groups in the pNIPAm chains. If the interaction between the composites is indeed governed by van der Waals forces, the cross-links could potentially be broken down by ultrasonication, which was observed experimentally. It was also found that the redispersed suspensions remained stable in solutions for multiple days despite the low zeta potential values (Figure 2d), which could be explained by steric stabilization imposed by the gels. The absence of composite stacking after redispersion indicated that the cross-linking is primarily driven above the VPTT of pNIPAm, suggesting that hydrophobic interactions may also play a role in the observed effect. Finally, the equilibrium size values after ultrasonication were found to be in the range of 240–300 nm, which was higher than both the free nanogels and titania particles, implying potential cohesion between the two.

**Figure 3:**
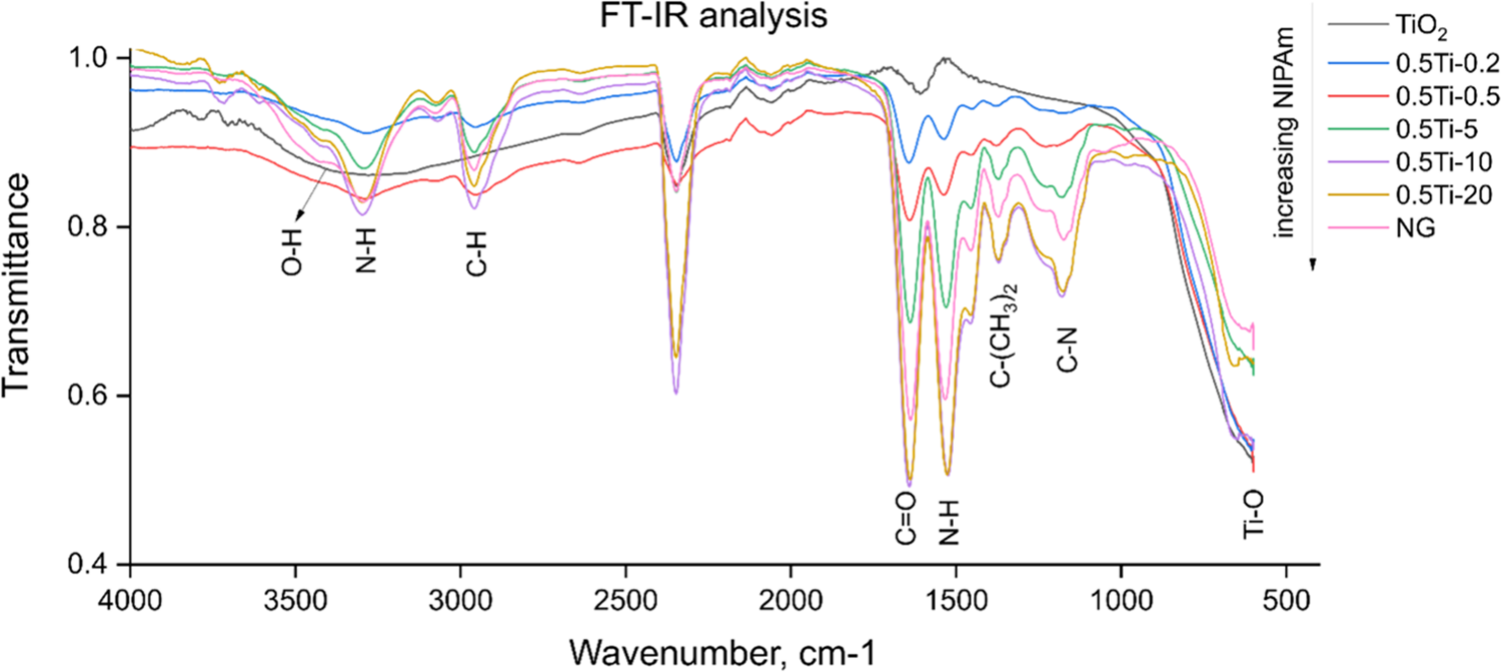
FT-IR spectra of TiO2-pNIPAm composites.

The chemical structure of the produced composites was further examined with the help of Fourier Transform Infrared (FT-IR) Spectroscopy as shown in Figure 3. The obtained spectra showed expected bands at 3500–3200 cm^–1^, 1600 and 1500 cm^–1^, 2800 and 1400 cm^–1^ arising from the N–H, C=O, C–H and C–N bonding respectively, matching well the anticipated structure of pNIPAm. Furthermore, one could see the representative bands of titania, namely at 3500–3000 cm^–1^ and 650 cm^–1^ representing Ti–OH and Ti–O–Ti bonds, respectively. There was a gradual evolution of the groups associated with pNIPAm with the increasing amount of monomer, while the peaks associated with TiO_2_ remained intense. This trend was especially clear when comparing the two peaks at 1645 and 1539 cm^–1^ corresponding to the secondary amide C=O stretching. In addition, there was an increase in absorbance for the 0.5Ti-10 and 0.5Ti-20 formulations suggesting a mixture of titania and pNIPAm. No additional vibration peaks were observed, thus confirming the purity of the samples.

### 3.2 VPTT and Volumetric Collapse

**Figure 4:**
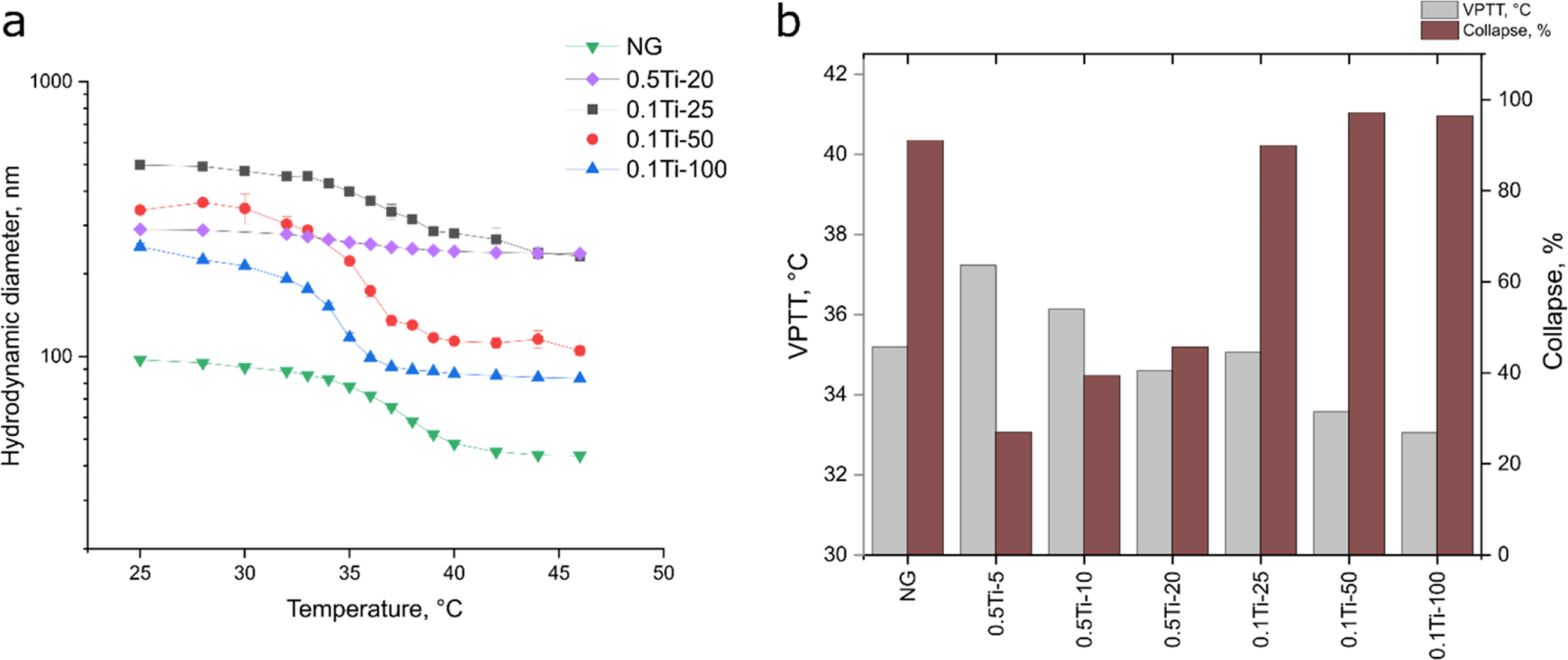
(a) Variation in size of different composite particles from 25 to 46 °C measured using Dynamic light scattering (DLS); (b) calculated VPTT and volumetric collapse values for different formulations.

The mechanism of volumetric phase transition temperature is well studied and is known to be driven by entropic release of bound water molecules due to the breakage of hydrogen bonds.^35^ Thus, polymer–polymer interactions become stronger than polymer–solvent interactions leading to phase separation and collapse of the NGs. It is important to note that due to the nature of the continuous phase transition, the system always exists on a spectrum between the collapsed or swollen states at any given temperature. Figure 4a depicts variations in particle size as a function of temperature. It is clear that the composite formulations with higher loading of titania interrupted the conventional sigmoidal size-temperature dependency and exhibited a curve flattening effect. This is particularly significant in the case of 0.5Ti-X formulations, where a lower percentage of free nanogel particles was expected. The reason behind the phenomenon is thought to be caused by the differences in morphology between the formulations. Titania nanoparticles act as ceramic cross-linkers that reduce gel flexibility. Furthermore, titania showed little to no deformation upon heating in the range of temperatures tested thus reducing the variations in the average particle size. Finally, it could help to maintain hydrogen bonds with the solvent, which, as a result, shifted the thermodynamic equilibrium toward a less-collapsed state at a given temperature. The size-temperature curves can also be used to extract the VPTT values as well as the corresponding degrees of volumetric collapse (Figure 4b). In situations where titania could potentially be encapsulated by the polymer, the particle shell layer was expected to collapse, with the core left unchanged. This would result in an effective decrease in the extent of volumetric collapse while retaining similar VPTT values seen for the pure nanogel since there would be little interaction between the particles and the solvent. There was, however, little effect on the 0.1Ti-X formulations with all values exceeding 90%, which was also observed for the bare nanogel. This suggested that the encapsulation efficiency was low with only the 0.1Ti-25 formulation exhibiting the predicted pattern with a clear curve flattening effect, as shown in Figure 4a. What is more, the VPTT values for the 0.1Ti-X appeared to be even lower than the reference case. This could be attributed to the fact that only the heating curves were captured, therefore hysteresis effects were not included in the calculation. On the contrary, the 0.5Ti-X formulations indicated a vastly different behavior suggesting both a higher titania loading as well as a different morphological pattern driving the VPTT values higher up. The overall increase in loading seemed to disrupt the polymer–solvent bonds giving rise to both a gel hardening effect and a significant increase in the VPTT inversely proportional to the amount of NIPAm. This pattern is expected to have implications on the emulsion droplet stability during the photocatalysis with the lower VPTT samples being more prone to droplet coalescence due to sample heating during irradiation. Further analysis is conducted in later chapters with an aim to establish correlations between phase behavior and the emulsion stability of the formulations.

### 3.3 Thermogravimetric (TGA) Analysis and TiO_2_ Loading

**Figure 5:**
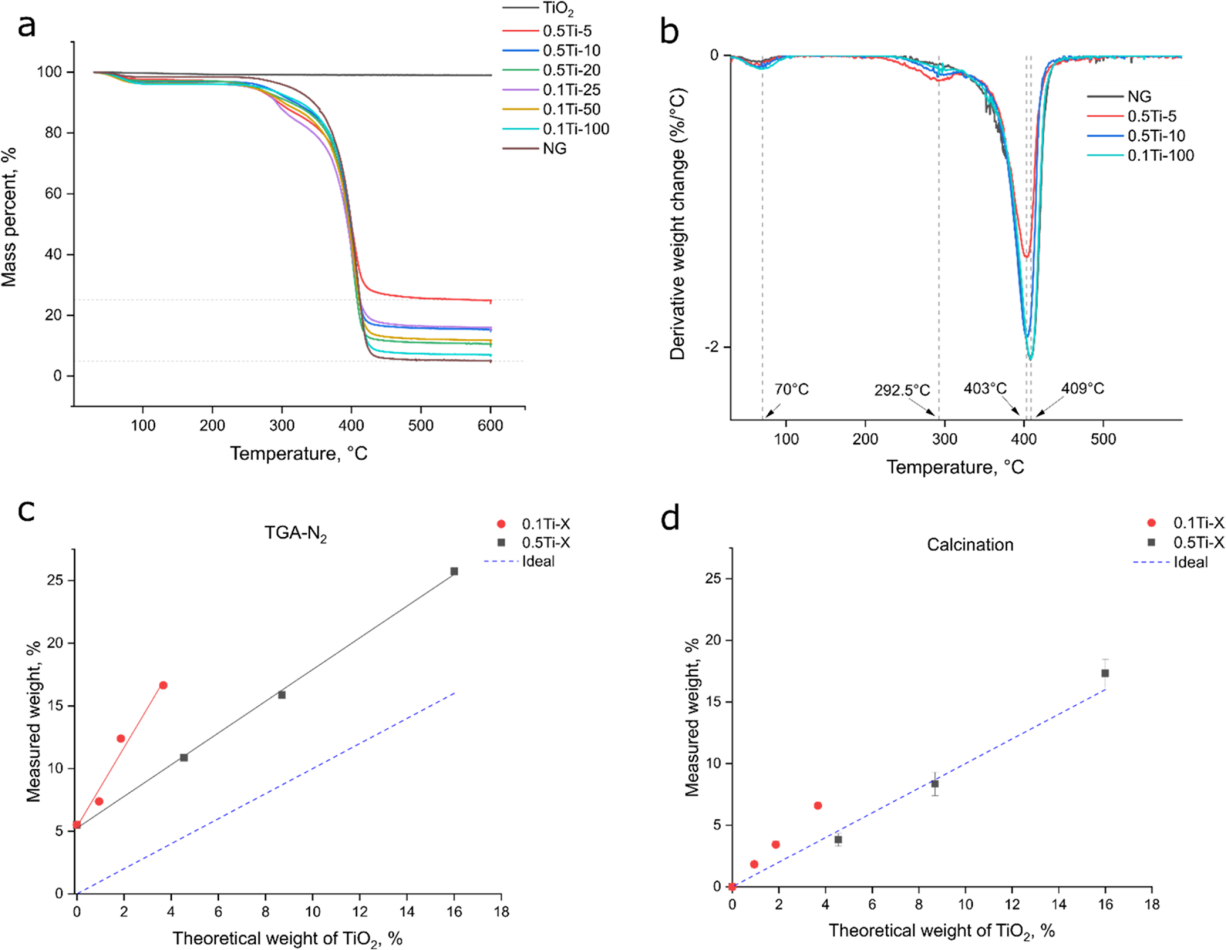
(a) TGA curves of the TiO_2_-pNIPAm composites, (b) DTG curves, (c) correlation between the predicted TiO_2_ loading versus the experimental data obtained from TGA, (d) TiO_2_ loading assessed by calcination.

In order to match the amount of titania in the formulations for photocatalytic testing, one has to quantify the loading of titania in the composites, since incomplete conversion gives rise to a higher weight fraction of titania. To assess the loading efficiency of titania in the produced composites as well as identify the polymer degradation characteristics arising from the inclusion of titania, TGA analysis was utilized. This was achieved by measuring the weight loss of the samples as a function of temperature. Figure 5a shows the degradation curves for some of the investigated formulations, whereas Figure 5b depicts the corresponding derivative thermogravimetric (DTG) curves. One can identify three main decomposition steps in the range of 30–100, 250–300, and 400–420 °C, respectively. The decrease in weight observed at lower temperature is attributed to the vaporization of physically adsorbed water and volatile substances. Interestingly, the second peak is only observed for the formulations containing titania, which suggests a synergetic effect arising from both pNIPAm and TiO_2_, which is enhanced by increasing the amount of titania in the formulations. Previous attempts have been made to characterize the thermal decomposition products of pNIPAm polymers during the third step with the suggested degradation products being volatile compounds from the pendant amide group such as carbon dioxide and water accompanied by nitrile compounds and imides.^36^ The main chain is thought to undergo random chain scission yielding gaseous oligomers of varying length. The remaining material is presumed to be elemental carbon since the powder can be easily oxidized by heating in ambient atmosphere. The overall profile was found to be similar for all the samples investigated with the main differences arising from the remaining mass percent values after the main step of decomposition, which is well correlated with the decreasing peak intensity of the DTG curves (Figure 5b). Furthermore, there was a slight shift in the main decomposition peak decreasing from 409 to 403 °C for the pure nanogel and the 0.5Ti-5 composite, respectively. This effect is in line with the general trend of favoring the composite decomposition at lower temperatures by introducing TiO_2_, as previously seen from the presence of the second peak of decomposition. The shift in decomposition temperature may be attributed to enhanced thermal conductivity in the composite from titania both facilitating heat transfer and introducing heterogeneity in the mesh, which could act as initiator sites for decomposition. The reference lines at higher temperatures indicate a range of values obtained for different samples, which vary from 5 to 25% for a bare nanogel and 0.5Ti-5, respectively. This is explained by the presence of titania, which exhibits a high thermal stability with only 2% weight loss under the same heating conditions.

When analyzing the residual masses obtained from the degradation profiles, one can see that there is a good correlation with the theoretical values of titania for the 0.5Ti-X samples (Figure 5c) differing mostly by the carbon offset. However, while the 0.1Ti-X samples demonstrate a linear increase in the measured values with respect to theoretical amount of TiO_2_, the slope of the curve is much higher than expected from the ideal case. This could be attributed to either an effect from incomplete conversion at low amounts of titania or as an enhanced carbonization of the polymer during the high-temperature steps of degradation. Furthermore, the effect seems to diminish when the higher concentration of titania is used in the polymerization mixture (0.5Ti-X). To get a better understanding of the process, a repeated decomposition experiment in ambient atmosphere was conducted (Figure 5d). In this case, the 0.5Ti-X formulations matched the theoretical loading of titania almost perfectly while a smaller yet significant deviation from the ideal curve was observed for the 0.1Ti-X formulations suggesting that the conversion after polymerization must have been lower than in the 0.5Ti-X case. Interestingly, the higher titania loading for the 0.1Ti-25 compared to 0.5Ti-20 matched well the sudden increase in the VPTT value (Figure 4b) showcasing the particle-loading – VPTT relationship.

### 3.4 Morphology Characterization

**Figure 6:**
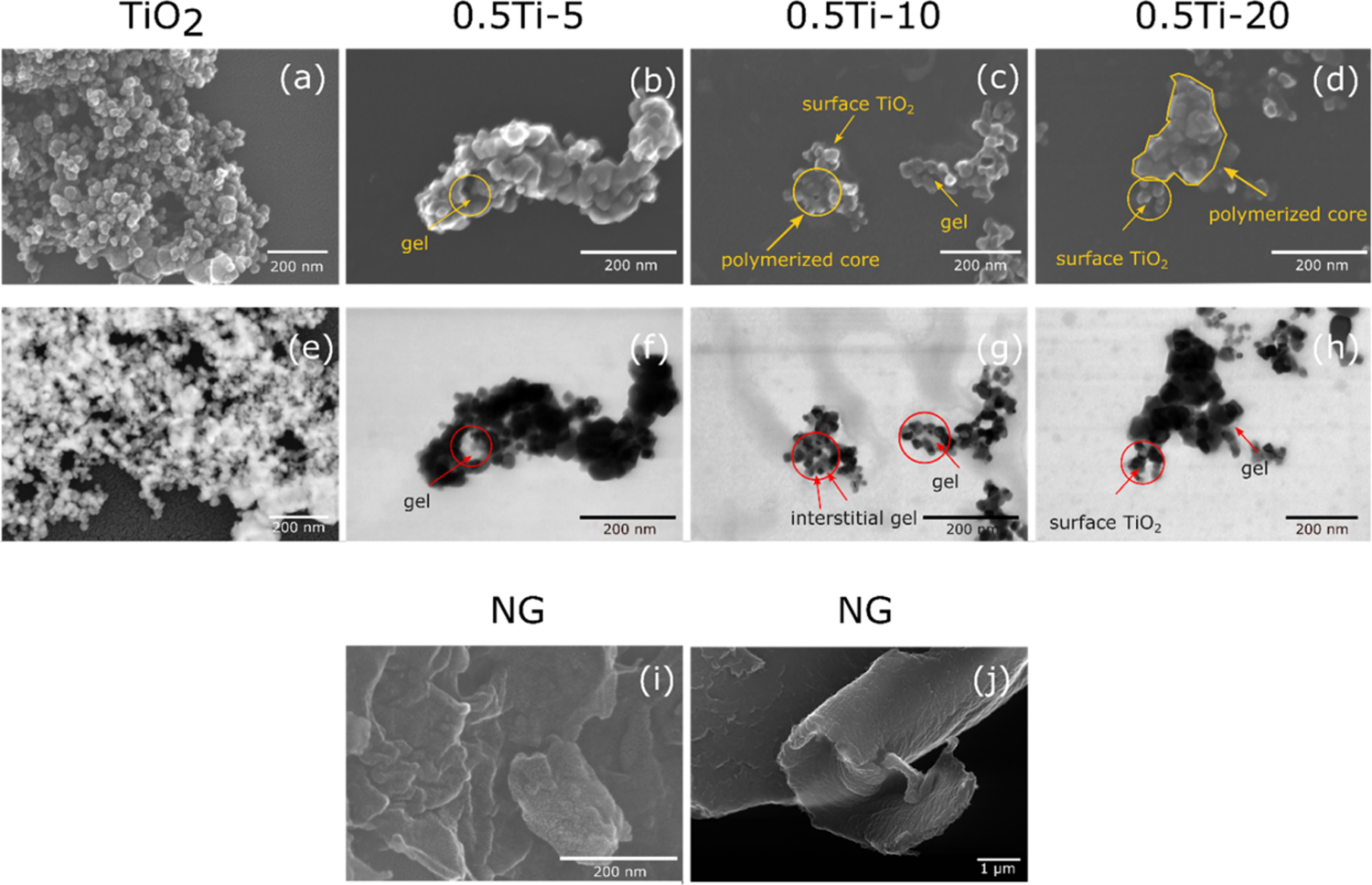
SEM images of titania nanoparticles (a), 0.5Ti-5 (b), 0.5Ti-10 (c), and 0.5Ti-20 composites (d); dark-field TEM image of titania nanoparticles (e) and bright-field TEM images of 0.5Ti-5 (f), 0.5Ti-10 (g), and 0.5Ti-20 composites (h); high (i) and low (j) magnification SEM images of the bare aggregated nanogels.

The size and morphology of the pristine titania particles and the synthesized TiO_2_-pNIPAm composites were analyzed using electron microscopy in both SEM and TEM modes (Figure 6). The TEM mode images have been taken to obtain an atomic number (Z) contrast that would help to make a distinction between the gels and titania as the lighter elements comprising pNIPAm scatter electrons weakly compared to titania.

The spherical titania primary particles were found to have an average size of 44 ± 25 nm (Figure 6a,e), whereas the irregularly shaped gels were in the range of 100 to 250 nm (Figure 6i). It is worth mentioning that bare titania was challenging to fully disperse and was always present in the flocculated form when analyzing the DLS results (∼200 nm, Figure 1b). In the case of the pure nanogels (NG) (Figure 6i,j), the obtained structure was composed of densely packed particles that form continuous sheets.

Regarding the composite samples (Figure 6b–d, f–h), a gradual increase in the amount of gel within the composites was clearly noted when looking at the samples from left to right. In the lowest NIPAm-to-particle ratio sample (Figure 6b), the composite appeared highly aggregated with only one area (marked by a circle), where the free pNIPAm gel could be observed. The presence of the pNIPAm gel was also confirmed by the TEM image (Figure 6f), which showed the gel as a dimmer surrounding matrix adjacent to the much darker titania. This configuration would then lead to the highest proportion of surface-exposed titania, potentially resulting in the greatest photocatalytic activity. When the amount of polymer was increased (Figure 6c), the composites exhibited a well-defined polymerized core containing trapped titania bound by the interstitial gels (Figure 6g). However, on the outside of these composites, one could still see titania, that was only partially covered by the gels, resembling the morphology of 0.5Ti-5. Finally, the 0.5Ti-20 sample (Figure 6d,h) showed a further increase in the size of the polymerized core leaving only a small fraction of surface-TiO_2_ directly exposed to irradiation. The observed trend correlated well with the relative amounts of titania and NIPAm in the reaction mixture leading to a higher proportion of gels in the composites for the samples with higher NIPAm concentrations. The resulting morphology pattern was expected to affect the photodegradation efficiency since the titania particles, trapped in the polymerized core, would be subject to both activation and pollutant diffusion limitations. Furthermore, when examining the VPTT and volumetric collapse data (Figure 4), a direct correlation between the morphology and the phase behavior of the composites was noted: pure nanogels exhibited a high degree of volumetric collapse, whereas the highly cross-linked (0.5Ti-5) composites demonstrated resistance to particle size changes upon heating resulting in higher VPTT and lower volumetric collapse values.

### 3.5 Emulsification Properties

The role of pNIPAm gels as wetting agents for titania was investigated through a partitioning study (Figure S6). In Figure S6a, a large portion of particles in the bare TiO_2_ formulation were not incorporated in the emulsion layer and stayed in the water phase. In contrast, both the 0.5Ti-10 and NG systems exhibited a high degree of incorporation into the emulsion phase, evidenced by much greater transparency in the water phase, especially in the 0.5Ti-10 sample. After aging for 48 h at room temperature, the bare TiO_2_ emulsion was fully demulsified, whereas the pNIPAm-based systems remained intact. When examining the droplet size distributions (Figure S6b), the increased translucency for the NG sample was attributed to tiny droplets that were less affected by gravity than the larger droplets produced with hybrid gels. Consequently, the hybrid emulsions could be almost instantaneously separated from the water phase, making the hybrid system highly effective for wastewater treatment applications.

To quantify particle partitioning between the oil and water phases, the water phase was retrieved and analyzed to determine the remaining particle concentration (Figure S6c). Since the bare TiO_2_ emulsions were fully demulsified, the titania concentration in the water phase matched the original concentration in the dispersion before emulsification. In contrast, the hybrid system showed only about 4% partitioning in the water phase, correlating with the visual appearance of the emulsions. For the bare NG emulsions, the degree of incorporation appeared to be similar to that of the hybrid particles; however, quantitative analysis was not possible due to challenges in layer separation and the low scattering intensity of the pNIPAm gels.

**Figure 7:**
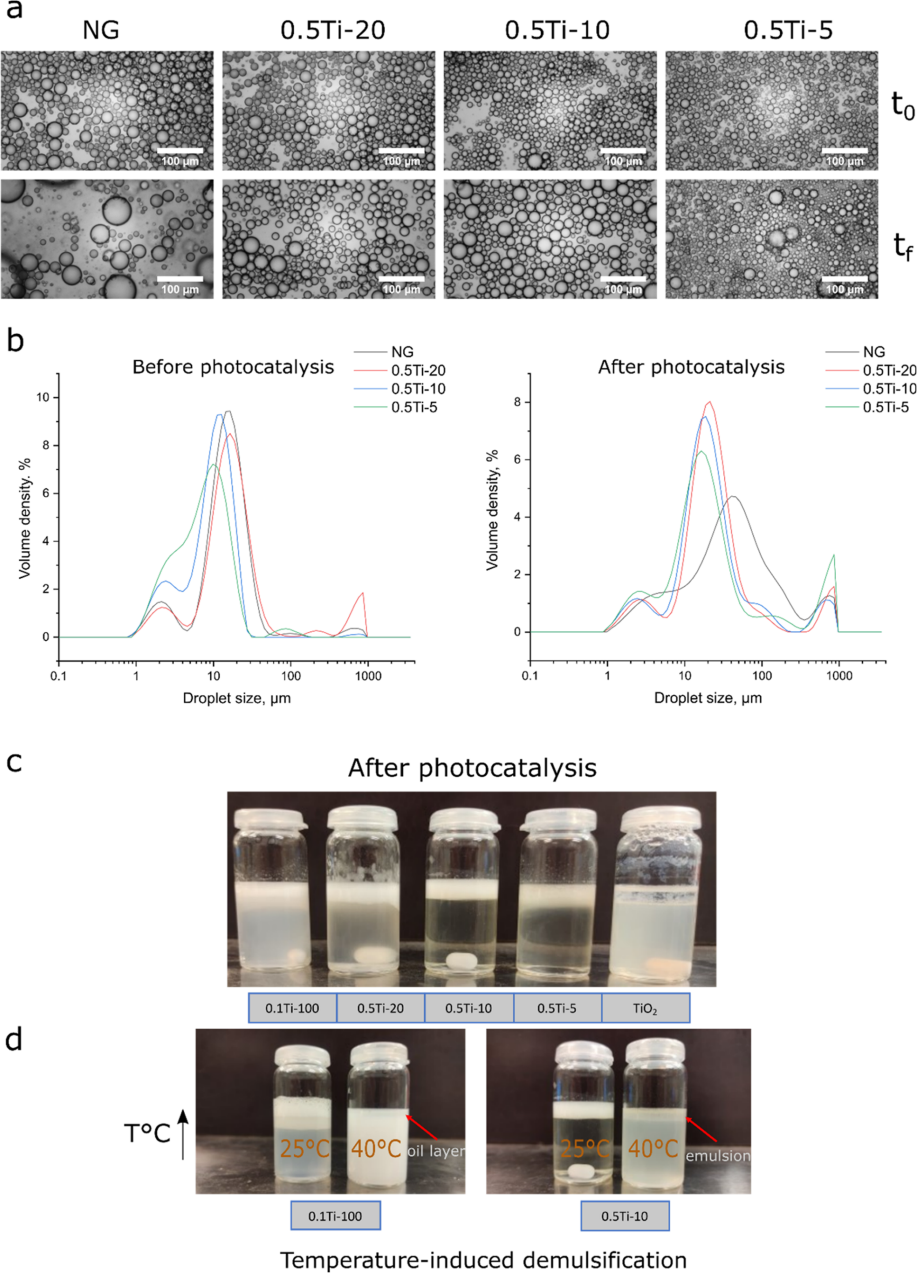
(a) Optical micrographs of the selected composites before (top) and after (bottom) photocatalytic degradation of pollutants, (b) the corresponding droplet size distributions, (c) photographs of the emulsions at the end of a photocatalytic cycle, (d) demulsification induced by heating emulsions above their VPTT.

The benefits of using composite particles as Pickering emulsifiers were also assessed by evaluating emulsion stability before and after the emulsions were subjected to a model wastewater system for a week under irradiation by a solar simulator. The emulsions were carefully mixed by ensuring an equal amount of titania in each of them. Figure 7a,b illustrate the droplet size distributions by optical microscopy and laser diffraction, respectively. These figures serve to evaluate the stability toward coalescence in the produced emulsions and visually depict the relative differences between the formulations. The TiO_2_-stabilized emulsion data were purposefully omitted due to its rapid coalescence immediately after mixing. When looking at the droplet size distributions before photocatalysis (Figure 7b), one can see a bimodal distribution ranging from 1 to 100 μm for the NG-stabilized emulsion, which then gradually shifts to smaller droplet sizes and approaches a single mode behavior as seen in the case of 0.5Ti-5. Despite the inherent hydrophilicity of pristine TiO_2_ particles, making it challenging to create stable Pickering emulsions, the observed trend reveals that a minimal quantity of polymer was needed to achieve emulsion stability. Overall, the peak volume density droplet sizes (Figure S5) indicate a relatively small variation between the distributions across the formulations.

After photodegradation, all distributions shifted toward larger droplet sizes, while the extent varied between the formulations. One can observe a high degree of coalescence for the NG sample, followed by slight flocculation observed for 0.5Ti-20, while the 0.5Ti-10 and 0.5Ti-5 formulations appeared to be quite stable despite a minor increase in droplet size. Since the temperature of the mixtures upon irradiation increases up to 32–35 °C, these differences are most likely associated with the temperature sensitivity and the extent of volumetric collapse for different particles as shown in Figure 4. In the case of NG, the VPTT temperature was relatively low, and the particle volumetric collapse was over 90%, which naturally increases the sensitivity of the resulting emulsions toward coalescence. Conversely, the TiO_2_-rich formulations had both a higher VPTT temperature and a much lower volumetric collapse, which led to decreased sensitivity toward temperature fluctuations. However, the decreased sensitivity reduced the stimuli-responsiveness, which was desired from the emulsion reusability point of view.

The digital images in Figure 7c depict the composite-stabilized emulsions after a single round of testing the photodegradation efficiency of 4-pb. It is evident that the composite-stabilized emulsions retained a high degree of integrity as an emulsion layer formed at the top of the samples after creaming, whereas a full phase separation was observed in the case of pure TiO_2_. The selected emulsions were then used to showcase the impact of heating pNIPAm-rich (0.1Ti-100) and TiO_2_-rich (0.5Ti-5) formulations above their VPTT temperature, thereby triggering demulsification (Figure 7d). It can be observed that in both cases, the mixtures became more turbid indicating an increased presence of particles in the aqueous phase. However, in the case of 0.5Ti-5, demulsification did not reach full completion, leaving a partially demulsified emulsion. This layer would need to be disposed of during recycling, thereby reducing the reusability potential for this formulation. Alternatively, the emulsion could be re-emulsified and reused by transferring the top emulsion layer from one pollutant solution to another. Overall, the process of emulsification and demulsification of emulsions stabilized by TiO_2_-pNIPAm particles was closely related to the phase transition behavior of the particles (Figure 4). Specifically, the composite emulsion could be emulsified at temperatures below the VPTT, demulsified at temperatures above the VPTT, and emulsified again when the temperature returned below the VPTT. It can be deduced that by raising the temperature above the VPTT value, the particles become more hydrophobic resulting in a contraction of particle size, which effectively opens free sites on the oil droplets thus making coalescence more likely upon droplet collision. This model is also backed by a temperature-induced flocculation observed for larger pNIPAm hydrogels, which has been analyzed on several occasions.^37,38^

### 3.6 Photocatalytic Degradation

**Figure 8:**
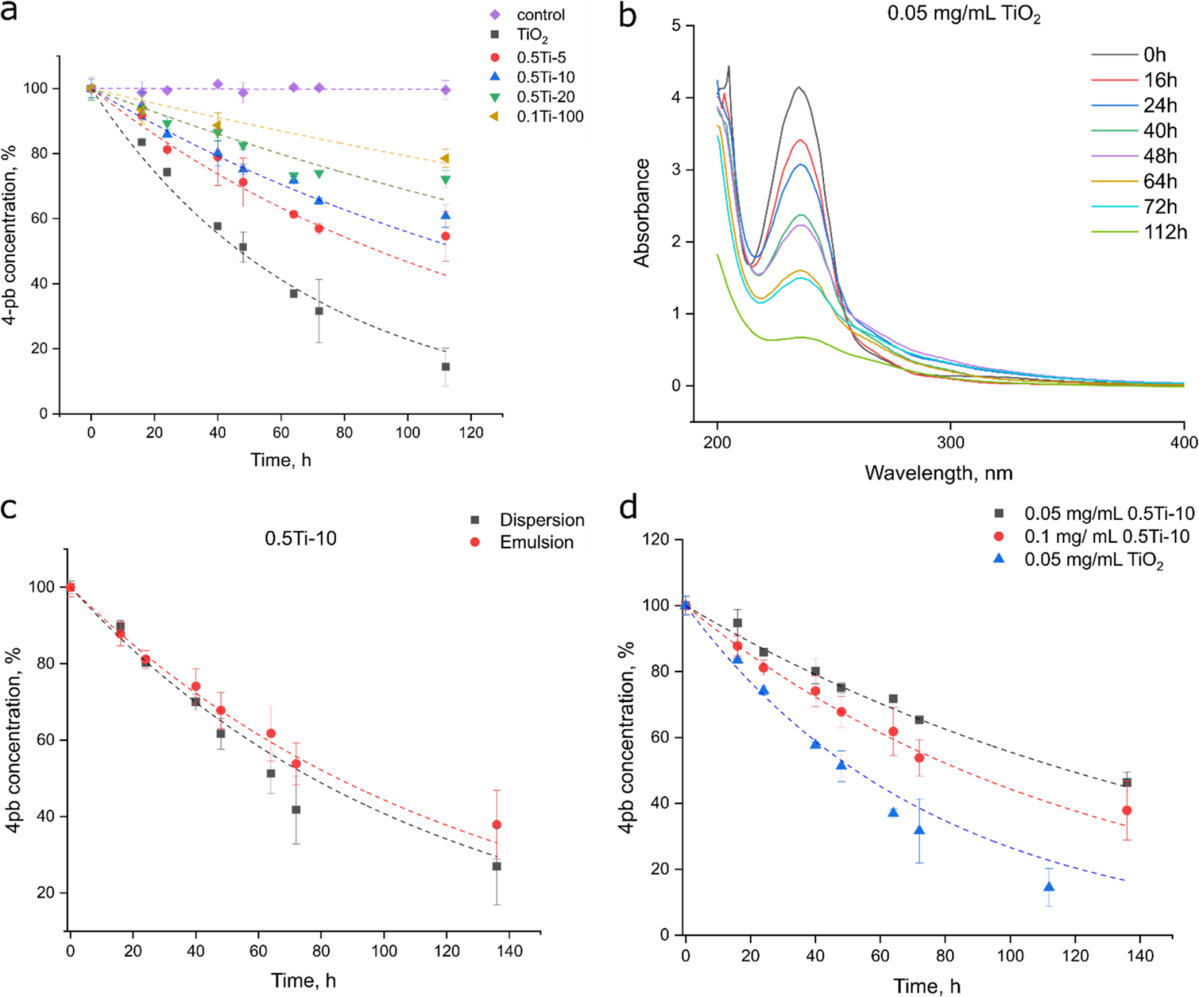
(a) Evolution of 4-pb concentration as a function of irradiation time by employing different TiO_2_-pNIPAm composites, (b) changes in the 4-pb solution absorbance as a function of photocatalytic irradiation time, (c) 0.5Ti-10 dispersion vs emulsion experiment, (d) 4-pb degradation as a function of composite concentration, bare TiO_2_ shown as a reference; the dotted lines represent the 1st order kinetic fitting.

The mechanism of photocatalytic degradation is known to take place via excitation of electrons to the conduction band, which undergo reduction of the dissolved oxygen to form superoxide anion radical (^•^O_2_^–^).^39^ Simultaneously, the formed holes in the valence band react with the adsorbed H_2_O/OH- generating hydroxyl radicals (^•^OH^–^). These radicals possess high redox potentials, leading to the oxidation of pollutants. In the case of 4-pb solutions, they turned yellow upon degradation indicating the presence of compounds featuring extended conjugated systems, like polycyclic aromatic hydrocarbons. However, further investigation is needed to confirm this. The photocatalytic efficiency of the produced composites was evaluated with respect to both pure titania-stabilized emulsions and the 4-pb control (without nanoparticles) under irradiation (Figure 8a). It is important to note that photocatalytic degradation of TiO_2_ dispersions was previously conducted showing analogous efficiency of the bare TiO_2_ emulsions since the produced emulsions are short-lived and effectively break down into a dispersion. Negligible degradation of the 4-pb control sample verified the effectiveness of the produced composite emulsions. The concentration change in the aliquots was calculated by following changes in absorbance of a distinct 4-pb peak at 235 nm (Figure 8b) and by using the corresponding calibration curve, shown in Figure S3. Since the peak position did not change with respect to 4-pb degradation, it was assumed that the resulting products either fully mineralized or produced compounds in the visible range which did not significantly affect the concentration-determination of 4-pb. Regarding the differences between the composites, one could see an increasing photocatalytic efficacy with respect to the loading of titania in the composites, which resembled the loading pattern seen in Figure 5d. Since the amount of titania was matched in the emulsions, the main differences arose from the morphology of the composite particles. For example, the 0.5Ti-5 formulation was found to have retained a high degree of titania particles in a form of flocs deposited on a gel surface (Figure 6b), thereby resulting in a relatively high photodegradation efficiency. In comparison, a slightly lower efficacy was observed with a lower density of titania particles with respect to the gel (0.5Ti-20). The degradation performance could be described by the pseudo-first-order kinetic model:
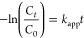
4where *C*_t_ and *C*_0_ denote the concentration of 4-pb at a certain timestamp and after attaining adsorption–desorption equilibrium, respectively, and *k*_app_ refers to the apparent pseudo-first-order kinetic constant (h^–1^). The derivation of the kinetic model is included in the Supporting Information file. The plot of ln(*C*_0_/*C*_t_) versus time shown in Figure S7 exhibited a good linear fit with the *k*_app_ values illustrated in Figure S8. It is worth noting that in the case of TiO_2_ – stabilized emulsion, the system coalesced almost instantaneously; therefore, the reported value can be considered to represent a TiO_2_ dispersion.

Given that ordered structures can be achieved in Pickering emulsions, a comparative study between the emulsified and dispersed variants of the composites was conducted by choosing the 0.5Ti-10 formulation as the model system. Furthermore, the composite concentration was doubled with respect to TiO_2_ to achieve both a higher extent of degradation during a similar period and to evaluate the potential for further optimization by increasing particle concentration. According to Figure 8c, there was an overlapping trend between the dispersed and emulsified systems within the first 40 h of irradiation, which then started to diverge and become more effective for the dispersed phase. The overall difference was rather small and could be attributed to the creaming of the emulsions, a gradual process over the course of irradiation. Furthermore, the reduction in performance coincided with the droplet flocculation observed at later timestamps, impeding the transport of 4-pb molecules to the surface of the droplets. Additionally, the emulsified systems appeared opaquer than the dispersed counterparts, giving rise to increased scattering and limited excitation of the titania particles present in the bulk. Later, the emulsion degradation data from the aforementioned study was combined with the values obtained during the formulation scanning experiments (Figure 8a) to display the changes in degradation kinetics as a function of particle concentration adjusted by TiO_2_ amount (Figure 8d). A blue dashed line representing the pattern of bare TiO_2_-emulsion was included as the reference. As shown from the graph, there was a notable increase in the slope of degradation by increasing the composite concentration from 0.05 to 0.1 mg/mL by TiO_2_. The obtained result indicated that one can achieve similar degradation kinetics to bare TiO_2_ by increasing the particle concentration in the emulsion while retaining enhanced stability and recyclability, characteristic of Pickering emulsions for photocatalytic applications. Further work on optimizing the photodegradation rate should be carried out, aiming to reduce the creaming and scattering of the composite emulsions by tuning the droplet size and oil concentration. This would provide a better light harvesting efficiency as well as provide high surface area for the photocatalytic degradation compared to dispersion systems. Furthermore, due to the large band gap of titania, the electron excitation from the valence band to the conduction gap is limited to photons in the UV range; hence, only a small portion of the solar spectrum is used to carry out pollutant degradation. This limitation could be overcome by forming hybrid nanoparticles, which would exhibit narrowing of the band gap and create trapping sites for the excited electrons, suppressing the recombination phenomenon.^40−42^

### 3.7 Emulsion Recovery

**Figure 9:**
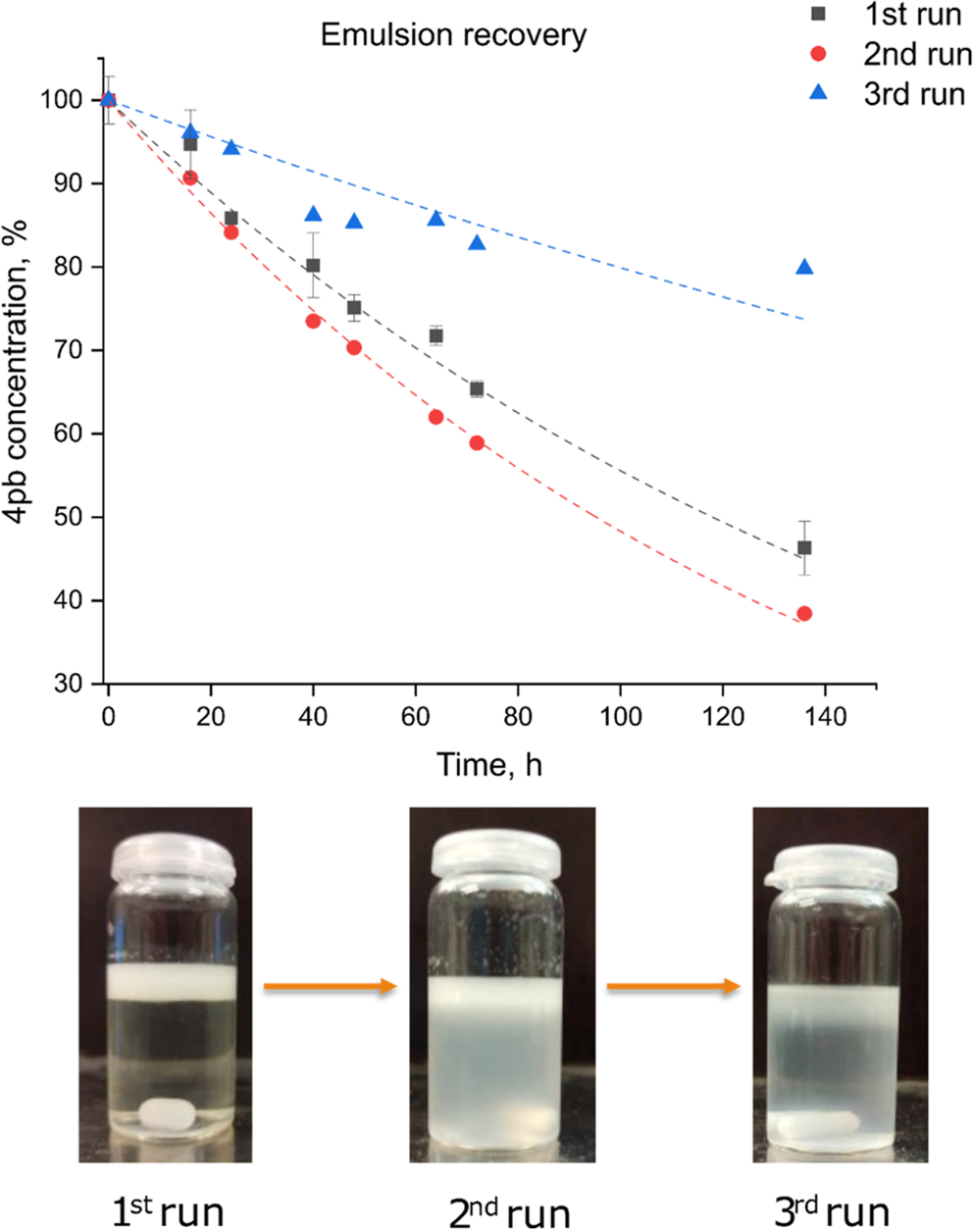
0.5Ti-10 emulsion photodegradation performance over 3 consecutive runs (above); the corresponding emulsion digital images after each run (below).

Having discussed the trends in photocatalytic degradation, it is important to assess the stability and reusability of the composite-stabilized emulsions over several cycles of photocatalysis. Contrary to nanoparticle dispersions, centrifugation after pollutant degradation is unnecessary, as the emulsions exhibit creaming behavior, facilitating separation from the treated water phase. Consequently, the water phase can be extracted by gravity separation while the emulsion layer is retained. To examine the reusability of the emulsions, three consecutive 4-pb degradation experiments with the spent 0.5Ti-10-stabilized emulsion were conducted after each run. Since the full creaming does not happen instantaneously, a fresh emulsion had to be partially replenished after each run to account for the losses when taking aliquots. As seen from Figure 9, high efficiency was maintained between the first two runs with the second run showing even a higher rate of organic acid decomposition. This could be attributed to either a partial emulsion breaking into a dispersion or the fluctuations in the projected light intensity on the sample during the experiment. However, during the third cycle, a substantial drop in the efficiency was observed accompanied by visually noticeable partial emulsion breaking. Despite the reported high efficiency of pNIPAm gels at stabilizing Pickering emulsions, some partitioning is expected, which limits the number of cycles the system can be reused. Given that dispersions were found to be slightly more effective at photodegradation compared to emulsions, demulsification was expected to enhance rather than diminish the performance. Therefore, the decreasing effectiveness was hypothesized to be caused by the degradation products being adsorbed on the particle surface, altering the surface wettability and leading to demulsification. The adsorbed species would block the surface sites on titania available for photodegradation, thus substantially reducing the degradation rate. The theory is supported by preliminary studies of 4-pb adsorption and the degradation products, which appeared to be more polar than the original 4-pb rendering them more water-soluble.

## 4 Conclusions

The study aims to lay the groundwork for future research on optimizing stimuli-responsive photocatalytic Pickering emulsions with the goal of developing innovative solutions for wastewater treatment. Specifically, this work reports a methodology for producing Pickering emulsions, stabilized by TiO_2_-pNIPAm gel composites with enhanced stability and easy recovery. The produced system shows potential for reusability while retaining a high degree of photocatalytic performance. Various aspects of particle and emulsion engineering have been discussed, including trends in VPTT, volumetric collapse, morphology, thermal stability and the corresponding photocatalytic degradation performance of a target naphthenic acid (4-pb). It was found that hydrogels coated with titania nanoparticles can be produced by tuning the amount of titania and the corresponding ratio between the monomer and the particles dispersed in the polymerization mixture when carrying out free radical polymerization. The differences in composite loading had direct implications for emulsion properties allowing them to be tuned accordingly. The optimal loading should balance emulsion stability and photocatalytic performance, as excessive particle coverage can hinder emulsification, while insufficient coverage diminishes photocatalytic efficiency. Furthermore, high loading of titania leads to an overall “stiffening” of the volumetric phase transition curve with increasing transition temperatures and decreasing degrees of volumetric collapse. This effect was found to have well-correlated implications in the temperature-driven demulsification of Pickering emulsions, stabilized by the composite particles. The elevated phase transition temperatures allowed to preserve high emulsion integrity when subjecting the systems to a model wastewater system for an extended period under irradiation by solar simulator. The emulsion recovery tests showed good reusability of the systems over multiple cycles of photodegradation. The photocatalytic efficiency was found to be lower for the composite systems compared to bare titania, which was postulated to be caused by reduced diffusivity of the analyte through the gel layers along with a lower particle excitation resulting from higher scattering. On the other hand, the pollutant degradation rate was found to depend on the composite concentration in the emulsion, allowing for potential optimization. Further studies have been proposed with the aim to rectify the drawbacks of the current formulations while retaining the benefits enlightened in this paper.

## Supporting Information Available

Emission spectrum of the SCIENCETECH SF300-A solar simulator; Tauc plots of citrate-stabilized titania; calibration curve used for 4-propylbenzoic acid (4-pb) determination; variation of zeta potential and particle diameter at different polymerization times from the aliquots of 0.1Ti-100; peak droplet sizes before and after 4-pb photodegradation; particle partitioning analysis of TiO_2_, 0.5Ti-10 and NG formulations; experimental data from the photocatalytic 4-pb degradation fitted to the first-order kinetics model; the apparent rate constants calculated from the first-order kinetics model; proposed photodegradation kinetics model (PDF)
