# Supplementary material for: Pickering Emulsions Stabilized by Hybrid TiO2-pNIPAm Composites for the Photocatalytic Degradation of 4-Propylbenzoic Acid
Source: ACS Omega. 2025 Jan 7;10(2):1988–2002. doi: 10.1021/acsomega.4c07847 (PMC11755169; doi:10.1021/acsomega.4c07847)
Supplement: Supplementary file 1 — ao4c07847_si_001.pdf [file ao4c07847_si_001.pdf]

Pickering emulsions stabilized by hybrid TiO_2-pNIPAm composites for the photocatalytic degradation of 4-propylbenzoic acid

Zygimantas Gricius and Gisle Øye*

* Corresponding author.

E-mail addresses: zygimantas.gricius@ntnu.no (Z. Gricius), gisle.oye@ntnu.no (G. Øye)

Ugelstad Laboratory, Department of Chemical Engineering, Norwegian University of Science and Technology (NTNU), 7491, Trondheim, Norway

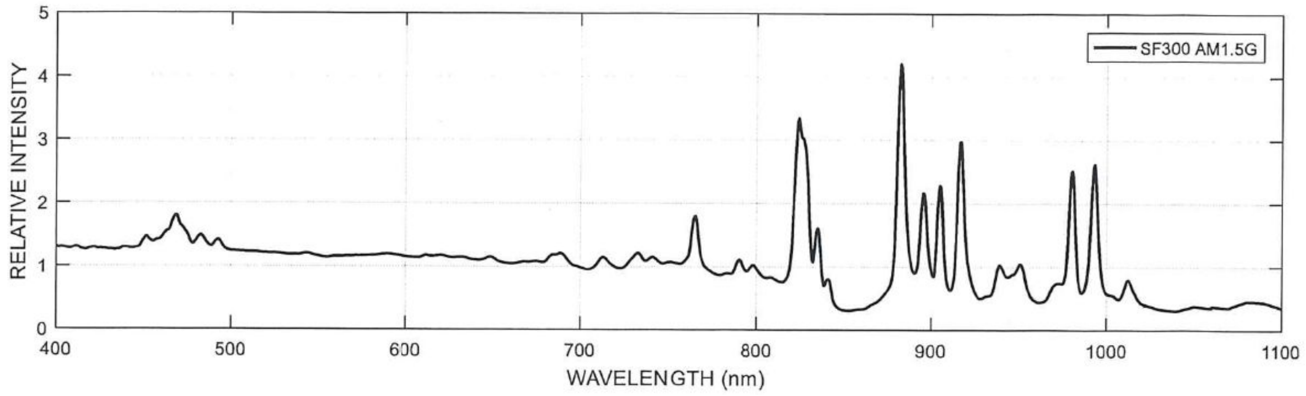

Figure S 1 Emission spectrum of the SCIENCETECH SF300-A solar simulator, reproduced from SCIENCETECH in accordance with ASTM E927-05 standards at an intensity of 1 sun.

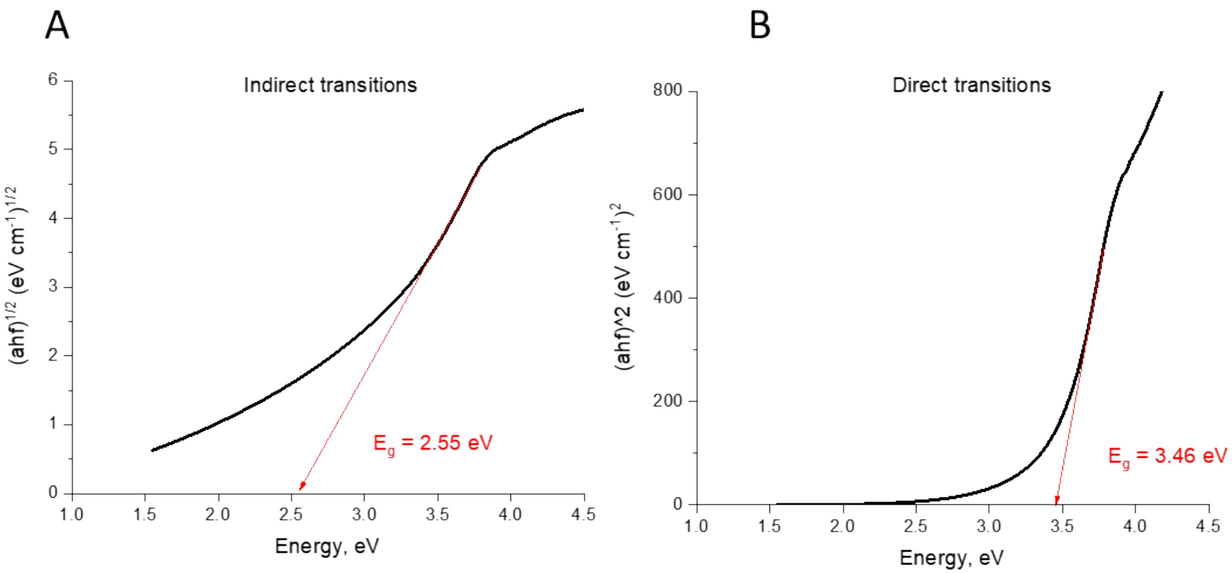

Figure S 2 Tauc plots of citrate-stabilized titania: A – indirect transitions, B – direct transitions

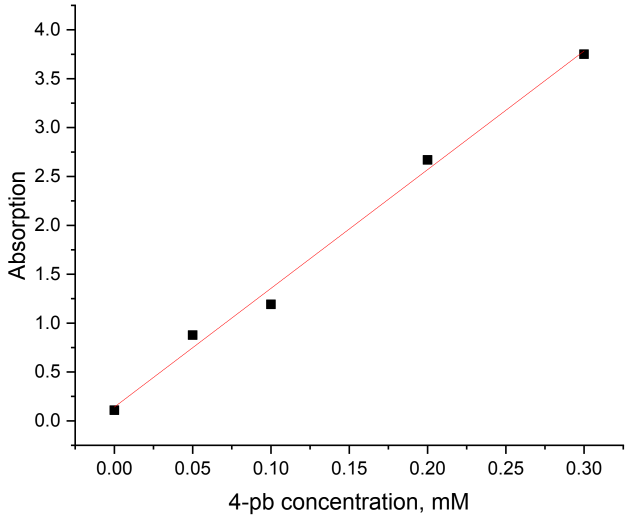

Figure S 3 Calibration curve used for 4-propylbenzoic acid (4-pb) determination. Absorption values taken at 235 nm.

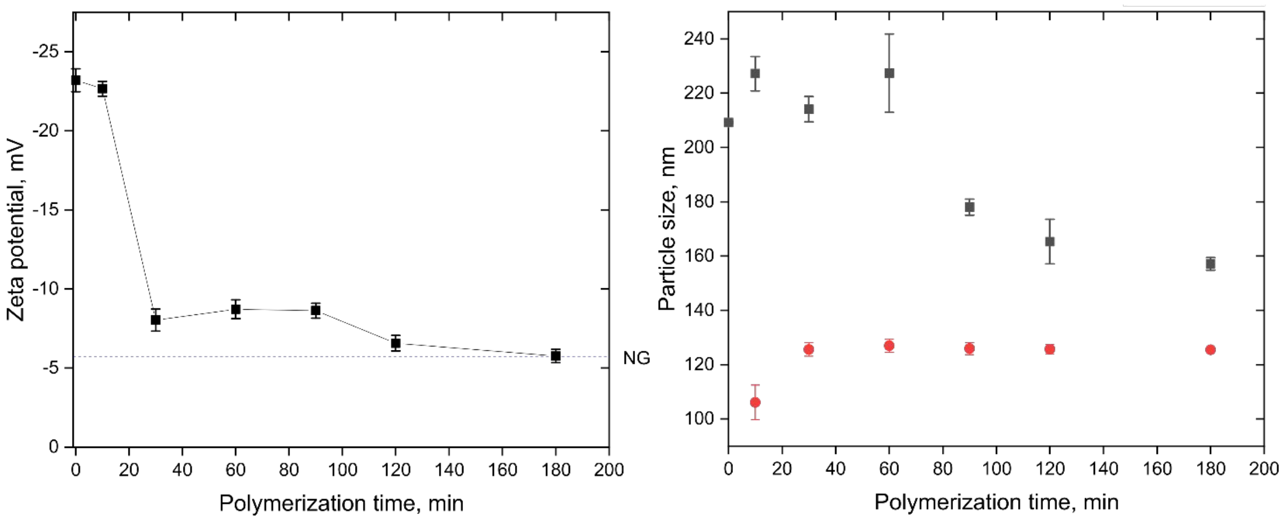

Figure S 4 Variation of zeta potential (left) and particle diameter (right) at different polymerization times from the aliquots of 0.1Ti-100

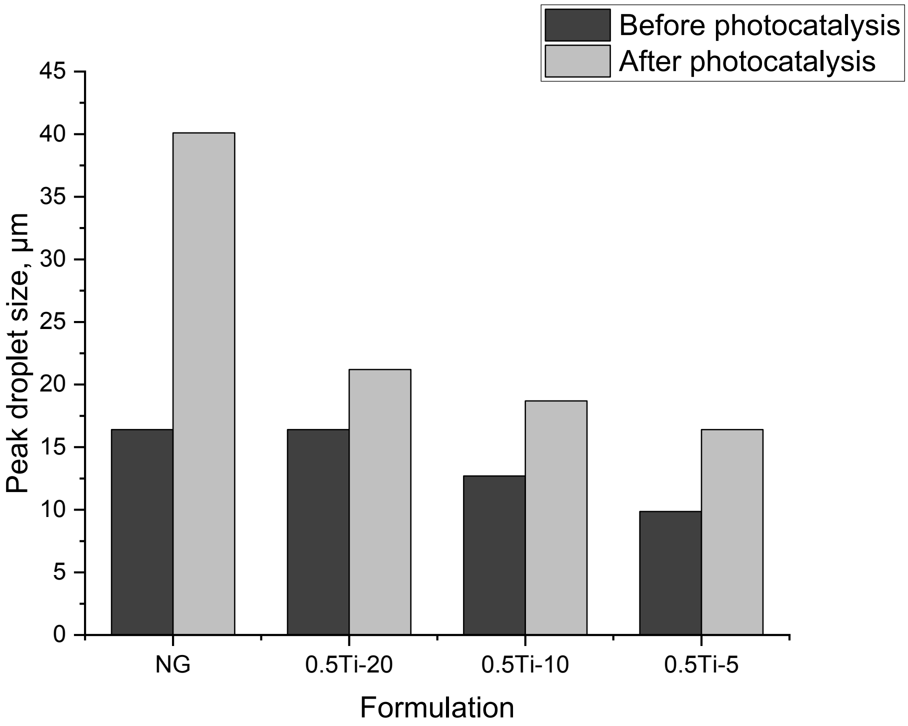

Figure S 5 Peak droplet sizes before and after 4-pb photodegradation

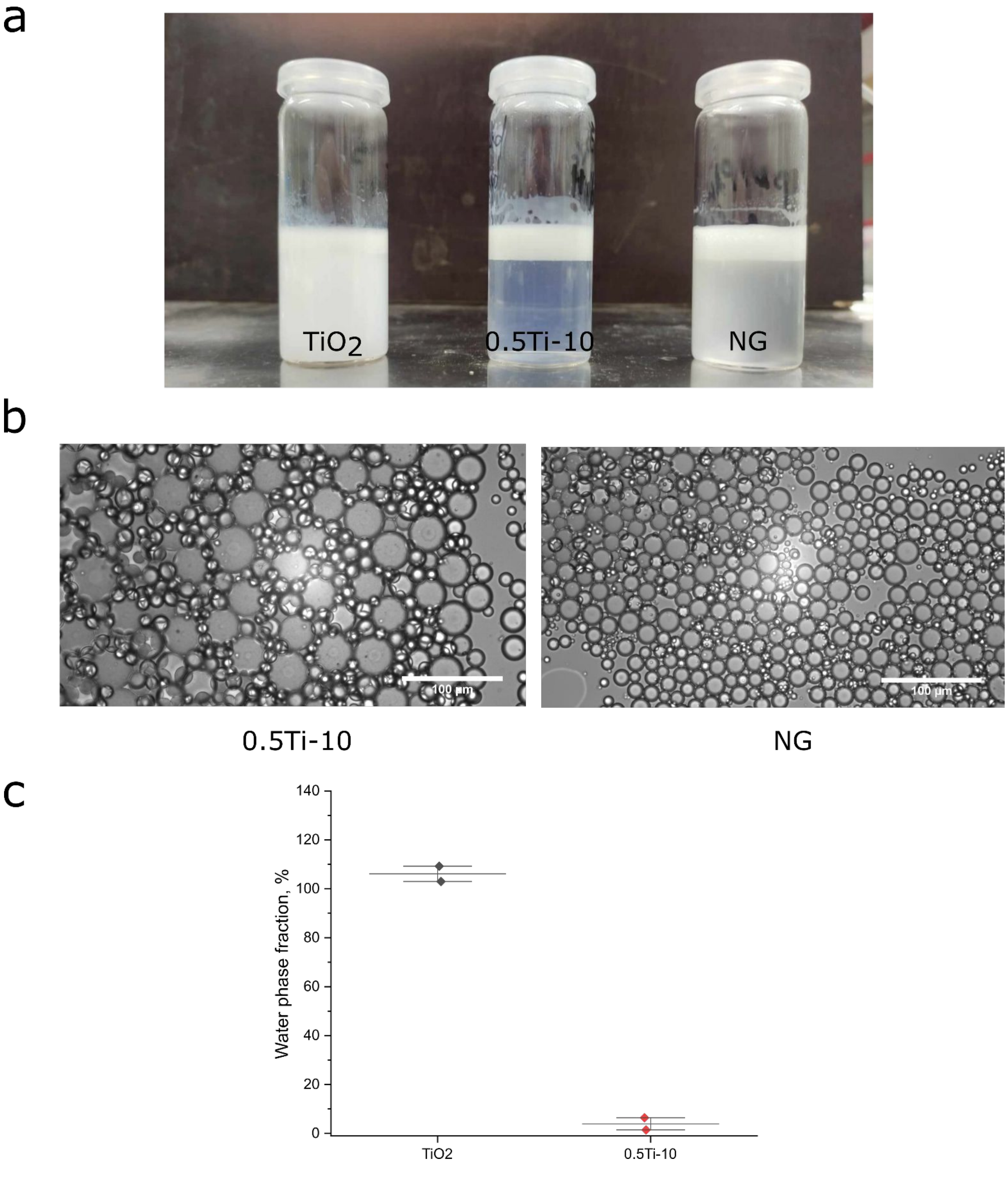

Figure S 6a - digital image of fresh TiO_2, 0.5Ti-10 and pure NG stabilized Pickering emulsions, b – the corresponding droplet size distributions, c – fractions of the particles left in the water phase after emulsification.

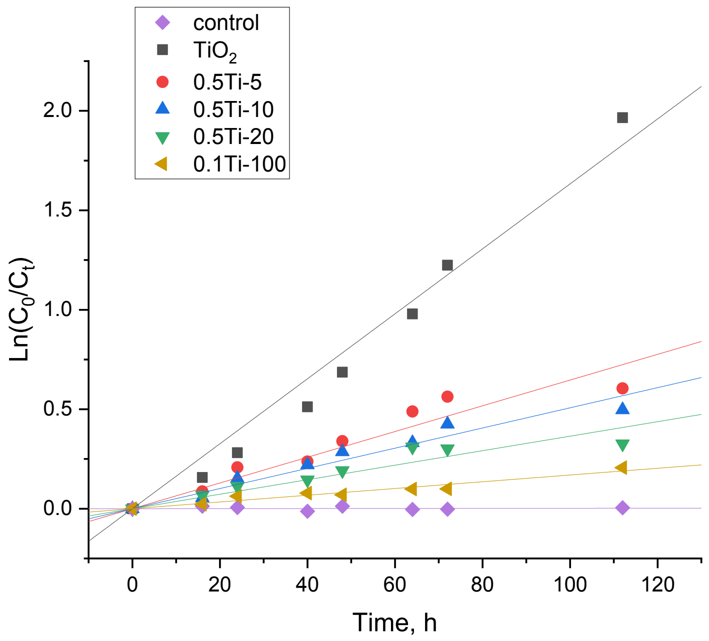

Figure S 7 Experimental data from the photocatalytic 4-pb degradation fitted to the first-order kinetics model.

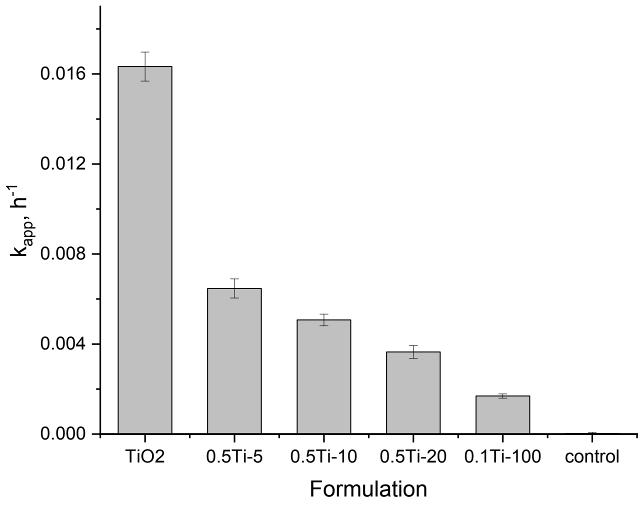

Figure S 8 The apparent rate constants calculated from the first-order kinetics model.

Proposed photodegradation kinetics model:

The mechanism of the photocatalysis involves three reactions: the adsorption of 4pb on an empty site of TiO_2 (1), the formation of the decomposition product which is assumed to be the Rate Determining Step (2) and the desorption of the product from TiO_2 (3). It can be assumed that 4pb decomposes irreversibility in reaction (2).

$$\text{(1)}\ \ 4\mathrm{pb} + \theta^* \leftrightarrow \theta_{4\mathrm{pb}}$$
$$\text{(2)}\ \ \theta_{4\mathrm{pb}} \rightarrow \theta_{\mathrm{p}}$$
$$\text{(3)}\ \ \theta_{\mathrm{p}} \leftrightarrow \theta^* + \mathrm{p}$$

The following rate equations are deducted, with r_+ the adsorption rate and r- the desorption rate:

$$\text{(4)}\ \ r = r_+ - r_- = K_{ads}C_{4pb}\theta^* - K_{des}\theta_{4pb}{}^*$$

$$\text{(5)}\ \ r_{RDS} = K_{RDS}\theta_{4pb}$$

$$\text{(6)}\ \ r = r_+ - r_- = K_{p,des}\theta_p - K_{p,ads}C_p\theta^*$$

By assuming a quasi-equilibrium in (4) meaning r=0, the concentration of 4pb can be expressed: $\theta_{4pb} = K_{4pb}C_{4pb}\theta^*$, with $K_{4pb} = \frac{K_{ads}}{K_{des}}$. By making the same assumption in (6), $\theta_p = K_pC_p\theta^*$, with $K_p = \frac{K_{p,ads}}{K_{p,des}}$, is obtained and knowing that the sum of all the sites is equal to 1, $\theta^* = \frac{1}{1+K_pC_p+K_{4pb}C_{4pb}}$ is obtained. θ^* is substituted in θ_{4pb} and θ_{4pb} is substituted in the RDS rate leading to $r_{RDS} = \frac{K_{RDS}K_{4pb}C_{4pb}}{1+K_pC_p+K_{4pb}C_{4pb}}$. This equation can be simplified through three cases, then integrated and plotted to express C_{4pb} as a function of time:

(7) The product adsorbs fast: $r_{RDS} = \frac{K_{RDS}K_{4pb}C_{4pb}}{1+K_{4pb}C_{4pb}}$

(8) The 4pb concentration is small: $r_{RDS} = K_{app}C_{4pb}$ with $K_{app} = K_{4pb}C_{4pb}$

→ $\ln\left(\frac{C_{4pb,0}}{C_{4pb}}\right) = K_{app}t$

(9) The 4pb concentration is large: $r_{RDS} = K_{RDS}$ → $C_{4pb} = C_{4pb,0} - K_{RDS}t$
